# Supplementary material for: What vaccination rate(s) minimize total societal costs after ’opening up’ to COVID-19? Age-structured SIRM results for the Delta variant in Australia (New South Wales, Victoria and Western Australia)
Source: PLOS Glob Public Health. 2022 Jun 14;2(6):e0000499. doi: 10.1371/journal.pgph.0000499 (PMC10021844; doi:10.1371/journal.pgph.0000499)
Supplement: S1 Text — (DOCX) [file pgph.0000499.s001.docx]

# S1 Text: Data sources

1. Daily data for total COVID19 cases, active, recovery, fatalities, fully vaccinated: https://www.covid19data.com.au (assessed 15/10/2021),
2. Daily data for hospitalisation, ICU admission, and ventilation requirements: https://covidlive.com.au/states-and-territories (assessed 15/10/2021)
3. Staffed ICU and ventilator capacity [21: box 1 - column 4, box 4 - column 5, box 3 - column 6]
4. Non-COVID19 (2018/2019) ICU bed days and ventilation rates: ANZICS. Intensive Care Resources and Activity Report 2018/19. Australian and New Zealand Intensive Care Society, 2020
5. Population data: https://www.abs.gov.au/statistics/people/population/national-state-and-territory-population/mar-2021/31010do002_202103.xls
