# Supplementary material for: What vaccination rate(s) minimize total societal costs after ’opening up’ to COVID-19? Age-structured SIRM results for the Delta variant in Australia (New South Wales, Victoria and Western Australia)
Source: PLOS Glob Public Health. 2022 Jun 14;2(6):e0000499. doi: 10.1371/journal.pgph.0000499 (PMC10021844; doi:10.1371/journal.pgph.0000499)
Supplement: S2 Text — Table A NSW: Hospitalization, ICU admissions, ventilation, and fatality. Table B Victoria: Hospitalization, ICU admissions, ventilation, and fatality. Table C Western Australia (the average of NSW and Victoria). (DOCX) [file pgph.0000499.s002.docx]

# S2 Text: Data-matched age distributions

Table A NSW: Hospitalization, ICU admissions, ventilation, and fatality

| Age group | Hospitalization | ICU admission | Ventilation | Fatality |
| --- | --- | --- | --- | --- |
| 0-9 | 2.552E-03 | 1.469E-04 | 7.160E-05 | 6.419E-05 |
| 10-19 | 7.229E-03 | 3.276E-04 | 1.597E-04 | 6.419E-05 |
| 20-29 | 3.375E-02 | 1.836E-03 | 8.950E-04 | 3.370E-04 |
| 30-39 | 9.257E-02 | 5.288E-03 | 2.578E-03 | 1.075E-03 |
| 40-49 | 1.307E-01 | 1.097E-02 | 5.346E-03 | 3.274E-03 |
| 50-59 | 3.038E-01 | 4.702E-02 | 2.293E-02 | 8.826E-03 |
| 60-69 | 5.021E-01 | 1.737E-01 | 8.468E-02 | 2.535E-02 |
| 70-79 | 7.337E-01 | 2.975E-01 | 1.450E-01 | 7.932E-02 |
| 80-89 | 7.976E-01 | 7.581E-02 | 3.696E-02 | 2.540E-01 |
| 90+ | 7.976E-01 | 7.581E-02 | 3.696E-02 | 4.599E-01 |

Table B Victoria: Hospitalization, ICU admissions, ventilation, and fatality

| Age group | Hospitalisation | ICU admission | Ventilation | Fatality |
| --- | --- | --- | --- | --- |
| 0-9 | 8.265E-04 | 5.159E-05 | 3.393E-05 | 4.996E-05 |
| 10-19 | 2.341E-03 | 1.151E-04 | 7.568E-05 | 4.996E-05 |
| 20-29 | 1.093E-02 | 6.449E-04 | 4.242E-04 | 2.623E-04 |
| 30-39 | 2.998E-02 | 1.857E-03 | 1.222E-03 | 8.369E-04 |
| 40-49 | 4.233E-02 | 3.852E-03 | 2.534E-03 | 2.548E-03 |
| 50-59 | 9.838E-02 | 1.652E-02 | 1.086E-02 | 6.870E-03 |
| 60-69 | 1.626E-01 | 6.101E-02 | 4.013E-02 | 1.974E-02 |
| 70-79 | 2.376E-01 | 1.045E-01 | 6.873E-02 | 6.174E-02 |
| 80-89 | 2.583E-01 | 2.663E-02 | 1.752E-02 | 1.977E-01 |
| 90+ | 2.583E-01 | 2.663E-02 | 1.752E-02 | 3.580E-01 |

Table C Western Australia (the average of NSW and Victoria)

| Age group | Hospitalisation | ICU admission | Ventilation | Fatality |
| --- | --- | --- | --- | --- |
| 0-9 | 1.689E-03 | 9.923E-05 | 5.277E-05 | 5.708E-05 |
| 10-19 | 4.785E-03 | 2.213E-04 | 1.177E-04 | 5.708E-05 |
| 20-29 | 2.234E-02 | 1.240E-03 | 6.596E-04 | 2.996E-04 |
| 30-39 | 6.127E-02 | 3.572E-03 | 1.900E-03 | 9.560E-04 |
| 40-49 | 8.652E-02 | 7.409E-03 | 3.940E-03 | 2.911E-03 |
| 50-59 | 2.011E-01 | 3.177E-02 | 1.689E-02 | 7.848E-03 |
| 60-69 | 3.324E-01 | 1.174E-01 | 6.241E-02 | 2.254E-02 |
| 70-79 | 4.856E-01 | 2.010E-01 | 1.069E-01 | 7.053E-02 |
| 80-89 | 5.280E-01 | 5.122E-02 | 2.724E-02 | 2.259E-01 |
| 90+ | 5.280E-01 | 5.122E-02 | 2.724E-02 | 4.090E-01 |
