# Supplementary material for: What vaccination rate(s) minimize total societal costs after ’opening up’ to COVID-19? Age-structured SIRM results for the Delta variant in Australia (New South Wales, Victoria and Western Australia)
Source: PLOS Glob Public Health. 2022 Jun 14;2(6):e0000499. doi: 10.1371/journal.pgph.0000499 (PMC10021844; doi:10.1371/journal.pgph.0000499)
Supplement: S3 Text — Table A NSW: Projected outcomes at different vaccination rates for opening up (1000’s people). Table B VIC: Projected outcomes at different vaccination rates for opening (1000’s people). Table B WA: Projected outcomes at different vaccination rates for opening up. (DOCX) [file pgph.0000499.s003.docx]

# S3 Text: Projected epidemiological outcomes

Table A NSW: Projected outcomes at different vaccination rates for opening up (1000’s people)

|  | Vaccination rates for opening up | | |
| --- | --- | --- | --- |
|  | 70% | 80% | 90% |
| Cumulative cases | 238.9  [225.2-252.3] | 203.8  [185.8-222.6] | 190.8  [171.1-210.6] |
| Peak active cases | 15.8  [14.7-17] | 12.4  [10.8-14] | 11  [9.2-12.9] |
| Peak hospitalization | 1.4  [1.3-1.5] | 1  [0.8-1.1] | 0.8  [0.7-0.9] |
| Peak ICU  (Capacity= 1.024; Average non-Covid demand=0.578; Net capacity=0.446) | 0.3  [0.3-0.3] | 0.2  [0.2-0.3] | 0.2  [0.2-0.2] |
| Peak ventilation  (Capacity=2.447; Average non-Covid demand=0.197; Net capacity=2.250) | 0.2  [0.1-0.2] | 0.1  [0.1-0.1] | 0.1  [0.1-0.1] |
| Cumulative fatalities | 1.9  [1.8-2] | 1.6  [1.4-1.7] | 1.4  [1.3-1.5] |

Notes:

1. Outside brackets are the mean, inside brackets are the 95% CI. Numbers are rounded to the nearest 1-decimal place.
2. ICU capacity was estimated at 1024 beds, including 884 available, staffed ICU beds plus 140 additional staffed ICU beds available. Average demand for ICU beds from non-COVID-19 patients was estimated at 578, i.e., ~211,000 bed days in 2018/19 (data sources reported in S1 Text). ICU net capacity (446 beds) is the difference between the total capacity and the non-COVID-19 demand.
3. Ventilation capacity includes 2,447 ventilators. Average demand for ventilators from non-COVID-19 patients is estimated at 197 in 2018/19, i.e., ~34% of the total admission (data sources reported in S1 Text). Ventilation net capacity (2,250 ventilators) is the difference between the total capacity and the non-COVID-19 demand.

Table B VIC: Projected outcomes at different vaccination rates for opening (1000’s people)

|  | Vaccination rates for opening up | | |
| --- | --- | --- | --- |
|  | 70% | 80% | 90% |
| Cumulative cases | 479.8  [424.4-533.2] | 360.6  [312.7-411.3] | 314.7  [271.4-357.9] |
| Peak active cases | 45.8  [38.8-53] | 30.1  [25-35.7] | 24  [19.8-28.4] |
| Peak hospitalization | 1.3  [1.1-1.5] | 0.8  [0.7-0.9] | 0.7  [0.7-0.7] |
| Peak ICU  (Capacity= 0.543; Average non-Covid demand=0.340; Net capacity=0.203) | 0.3  [0.3-0.4] | 0.2  [0.2-0.2] | 0.1  [0.1-0.2] |
| Peak ventilation  (Capacity=2.023; Average non-Covid demand=0.141; Net capacity=1.882) | 0.2  [0.2-0.2] | 0.1  [0.1-0.1] | 0.1  [0.1-0.1] |
| Cumulative fatalities | 3.5  [3.2-3.8] | 2.7  [2.4-2.9] | 2.3  [2.1-2.5] |

Notes:

1. Outside brackets are the mean, inside brackets are the 95% CI. Numbers are rounded to the nearest 1-decimal place.
2. ICU capacity is estimated at 543 beds, including 476 available, staffed ICU beds plus 67 additional staffed ICU beds available. Average demand for ICU beds from non-COVID-19 patients is estimated at 340, i.e., ~124,000 bed days in 2018/19 (data sources reported in S1 Text). ICU net capacity (203 beds) is the difference between the ICU capacity and non-COVID-19 demand.
3. Ventilation capacity is estimated at 2,203. Average demand for ventilators from non-COVID-19 patients is estimated at 141 in 2018/19, i.e., ~42% of the total admission (data sources reported in S1 Text). Ventilation net capacity (1,882 ventilators) is the difference between the ventilation capacity and non-COVID-19 demand.

Table C WA: Projected outcomes at different vaccination rates for opening up

|  | Vaccination rates for opening up | | |
| --- | --- | --- | --- |
|  | 70% | 80% | 90% |
| Cumulative cases (rounded to the nearest 1000) | 31.2  [28.2-35.4] | 11  [10-12.1] | 4.4  [4.1-4.8] |
| Peak active cases (rounded to the nearest 1000) | 3.6  [3.2-4.2] | 1.2  [1-1.4] | 0.4  [0.4-0.5] |
| Peak hospitalization (people) | 164  [143-189] | 54  [47-61] | 18  [16-21] |
| Peak ICU (people)  (Capacity= 190; Average non-Covid demand=102; Net capacity=88) | 40  [35-47] | 13  [12-15] | 5  [4-5] |
| Peak ventilation (people)  (Capacity=518; Average non-Covid demand=42; Net capacity=476) | 22  [19-25] | 7  [6-8] | 2  [2-3] |
| Cumulative fatalities (people) | 191  [173-215] | 68  [62-74] | 29  [27-30] |

Notes:

1. Outside brackets are the mean, inside brackets are the 95% CI. Numbers are rounded to the nearest 1-decimal place.
2. ICU capacity is estimated at 190 beds, including 159 available, staffed ICU beds plus 31 additional staffed ICU beds available. Average demand for ICU beds from non-COVID-19 patients is estimated at 102, i.e., ~37,300 bed days in 2018/19 (data sources reported in S1 Text). ICU net capacity (88 beds) is the difference between the ICU capacity and non-COVID-19 demand.
3. Ventilation capacity is estimated at 518. Average demand for ventilators from non-COVID-19 patients is estimated at 42 in 2018/19, i.e., ~43% of the total admission (data sources reported in S1 Text). Ventilation net capacity (476 ventilators) is the difference between the ventilation capacity and non-COVID-19 demand
