# Supplementary material for: What vaccination rate(s) minimize total societal costs after ’opening up’ to COVID-19? Age-structured SIRM results for the Delta variant in Australia (New South Wales, Victoria and Western Australia)
Source: PLOS Glob Public Health. 2022 Jun 14;2(6):e0000499. doi: 10.1371/journal.pgph.0000499 (PMC10021844; doi:10.1371/journal.pgph.0000499)
Supplement: S4 Text — Table A NSW: Estimates of health care and welfare losses, billions AUD. Table B VIC: Estimates of health care and welfare losses, billions AUD. Table C WA: Projected health care costs and welfare losses, millions AUD. (DOCX) [file pgph.0000499.s004.docx]

# S4 Text: Estimates of health care and welfare losses at different vaccination rates

Table A. NSW: Estimates of health care and welfare losses, billions AUD

| Vaccination rate | Health care services | | | Welfare losses | | | Total Health care and welfare losses |
| --- | --- | --- | --- | --- | --- | --- | --- |
|  | Non-ICU | ICU | Total | Recover people | Non-recovered | Total |  |
| 70% | 0.3  [0.2-0.3] | 0.2  [0.2-0.2] | 0.5  [0.4-0.5] | 2.3  [2.1-2.6] | 3  [2.8-3.2] | 5.3  [4.9-5.8] | 5.8  [5.3-6.3] |
| 80% | 0.2  [0.2-0.2] | 0.2  [0.1-0.2] | 0.4  [0.3-0.4] | 1.9  [1.6-2.1] | 2.3  [2-2.6] | 4.2  [3.6-4.8] | 4.5  [3.9-5.2] |
| 90% | 0.2  [0.2-0.2] | 0.1  [0.1-0.2] | 0.3  [0.3-0.4] | 1.7  [1.4-2] | 2  [1.7-2.3] | 3.7  [3.1-4.3] | 4  [3.4-4.7] |

Notes:

1. All costs are counted from 11 October 2021.
2. Outside brackets are the mean, inside brackets are the 95% CI. Numbers are rounded to the nearest 1-decimal place.

Table B. VIC: Estimates of health care and welfare losses, billions AUD

| Vaccination rate | Health care services | | | Welfare losses | | | Total Health care and welfare losses |
| --- | --- | --- | --- | --- | --- | --- | --- |
|  | Non-ICU | ICU | Total | Recover people | Non-recovered | Total |  |
| 70% | 0.2  [0.2-0.2] | 0.2  [0.2-0.2] | 0.4  [0.3-0.4] | 5.9  [5.1-6.7] | 5.6  [4.9-6.3] | 11.5  [10-13] | 11.9  [10.3-13.5] |
| 80% | 0.1  [0.1-0.2] | 0.1  [0.1-0.1] | 0.3  [0.2-0.3] | 4.3  [3.6-5] | 3.9  [3.3-4.5] | 8.2  [7-9.5] | 8.5  [7.2-9.8] |
| 90% | 0.1  [0.1-0.1] | 0.1  [0.1-0.1] | 0.2  [0.2-0.3] | 3.7  [3.1-4.3] | 3.1  [2.7-3.6] | 6.8  [5.8-7.9] | 7.1  [6-8.2] |

Notes:

1. Only costs after 11/10/2021 are counted.
2. Outside brackets are the mean, inside brackets are the 95% CI. Numbers are rounded to the nearest 1-decimal place.

Table C. WA: Projected health care costs and welfare losses, millions AUD

| Vaccination rate | Health care services | | | Welfare losses | | | Total Health care and welfare losses |
| --- | --- | --- | --- | --- | --- | --- | --- |
|  | Non-ICU | ICU | Total | Recover people | Non-recovered | Total |  |
| 70% | 27  [23-30] | 23  [20-26] | 50  [44-57] | 399  [353-456] | 408  [366-459] | 807  [719-915] | 857  [763-971] |
| 80% | 9  [8-10] | 7  [7-8] | 16  [14-18] | 130  [116-147] | 131  [119-146] | 262  [235-293] | 278  [249-311] |
| 90% | 3  [3-3] | 2  [2-3] | 5  [5-6] | 44  [39-49] | 44  [39-49] | 88  [78-98] | 93  [83-104] |

Notes:

1. Only counting costs after 11 October 2021.
2. Outside brackets are the mean, inside brackets are the 95% CI. Numbers are rounded to the nearest million dollars.
