# Supplementary material for: What vaccination rate(s) minimize total societal costs after ’opening up’ to COVID-19? Age-structured SIRM results for the Delta variant in Australia (New South Wales, Victoria and Western Australia)
Source: PLOS Glob Public Health. 2022 Jun 14;2(6):e0000499. doi: 10.1371/journal.pgph.0000499 (PMC10021844; doi:10.1371/journal.pgph.0000499)
Supplement: S5 Text — Table A NSW: Per capita (AUD and %) differences from Opening Up at alternative vaccination rate to the preferred vaccination rate (80%). Table B NSW: Sensitivity of preferred vaccination rate to the effectiveness of public health measures after opening up at reducing community transmission and the speed of vaccination. Table C NSW: Sensitivity of preferred vaccination rate to economy lockdown cost and community transmission during lockdown. Table D VIC: Per capita (AUD and %) cost differences of alternative vaccination rates to the preferred vaccination rate (90%). Table E VIC: Sensitivity of the preferred vaccination rate to the effectiveness of public health measures after opening up and speed of vaccination. Table F VIC: Sensitivity of preferred vaccination rate to economy lockdown costs and rate of community transmission during lockdown Table G WA: Per capita (AUD and %) cost differences of alternative vaccination rates to the preferred vaccination rate (89%). Table H WA: Sensitivity of preferred vaccination rate to the effectiveness of public health measures after opening up at reducing community transmission and speed of vaccination. Table I WA: Sensitivity of preferred vaccination rate to opening up with the probability of seeding and state border closure costs. (DOCX) [file pgph.0000499.s005.docx]

# S5 Text: Sensitivity Analysis

Table A NSW: Per capita (AUD and %) differences from Opening Up at alternative vaccination rate to the preferred vaccination rate (80%)

| Vaccination rate | Health care and welfare loss AUD/person | Lockdown Economy cost | Total cost |
| --- | --- | --- | --- |
| 70% | +158 (+28.1%) | -99 (-100%) | +59 (+8.9%) |
| 80% | 0 (0%) | 0 (0%) | 0 (0%) |
| 90% | -66 (-11.8%) | +99 (+100%) | +33 (+5%) |

Notes:

1. Numbers are per-capita deviations from the economically justifiable vaccination rate, '+' = higher and '-' =lower.
2. All numbers are counted from 11/10/2021
3. Outside brackets are the absolute value of the deviation rounded to the nearest $/person
4. Inside brackets are the relative deviation in percentage rounded to the nearest 1-decimal place.

Table B NSW: Sensitivity of preferred vaccination rate to the effectiveness of public health measures after opening up at reducing community transmission and the speed of vaccination

|  | | Daily vaccination rate  (*Baseline =64,000 people/day*) | | |
| --- | --- | --- | --- | --- |
|  |  | Baseline | 110% baseline | 120% baseline |
| Reduction in community transmission after Opening Up (*Baseline optimistic level =30% reduction in community transmission with public health measures)* | 20% | >90% (14.8) | >90% (14.6) | >90% (13.1) |
|  | 25% | 89% (8.4) | 89% (8.3) | 88% (7.5) |
|  | 30% | 80% (5.4) | 80% (5.3) | 79% (4.9) |

Notes:

1. Outside brackets are the preferred vaccination rate to opening up.
2. Inside brackets are the associated total societal cost from October 11^th^ 2021 in billion dollars, rounded to the nearest 1-decomal point associated with preferred vaccination rates.

Table C NSW: Sensitivity of preferred vaccination rate to economy lockdown cost and community transmission during lockdown

|  | | Lockdown cost compared to low-level restriction cost  *Baseline level = (2.35-0.65) X 31.7% billion AUD/week* | | |
| --- | --- | --- | --- | --- |
|  |  | 80% baseline | Baseline | 120% baseline |
| Daily community transmission during lockdown  (*Baseline level =14-day average prior to 11/10/2021*) | 80% baseline | 84%  (4.7) | 82%  (4.9) | 80%  (5.1) |
|  | Baseline | 83%  (5.2) | 80%  (5.4) | 78%  (5.5) |
|  | 120% baseline | 78%  (5.7) | 75%  (5.8) | 71%  (5.9) |

Notes:

1. Outside brackets are the economically justifiable vaccination target for removing the lockdown.
2. Inside brackets are the associated total cost from October 11, 2021 in billion dollars, rounded to the nearest 1-decimal place.

Table D VIC: Per capita (AUD and %) cost differences of alternative vaccination rates to the preferred vaccination rate (90%)

| Vaccination rate | Health care Costs and Welfare Losses | Economy Lockdown Cost | Total cost |
| --- | --- | --- | --- |
| 70% | +723 (+68.2%) | -214 (-58.1%) | +508 (+35.6%) |
| 80% | +211 (+19.9%) | -107 (-29.1%) | +104 (+7.3%) |
| 90% | 0 (0%) | 0 (0%) | 0 (0%) |

Notes:

1. Numbers are per-capita deviations from the economically justifiable vaccination rate, '+' = higher and '-' =lower
2. Outside brackets are the absolute value of the deviation rounded to the nearest $/person.
3. Inside brackets are the relative deviation in percentage rounded to the nearest 1-decimal place.

Table E VIC: Sensitivity of the preferred vaccination rate to the effectiveness of public health measures after opening up and speed of vaccination

|  | | Daily vaccination rate  (Baseline =45,000 people/day) | | |
| --- | --- | --- | --- | --- |
|  |  | Baseline | 110% baseline | 120% baseline |
| Reduction in community transmission after Opening Up (*Baseline optimistic level =30% reduction in community transmission with public health measures)* | 20% | >90% (17.3) | >90% (17.1) | >90% (16.8) |
|  | 25% | >90% (12.3) | >90% (12.3) | >90% (11.7) |
|  | 30% | 90%  (9.6) | 90%  (9.3) | 90%  (9.0) |

Notes:

1. Outside brackets are the economically justifiable vaccination target for removing the lockdown.
2. Inside brackets is the associated total cost from October 11^th^ 2021 in billion dollars, rounded to the nearest 1-decimal place.

Table F VIC: Sensitivity of preferred vaccination rate to economy lockdown costs and rate of community transmission during lockdown

|  | | Lockdown cost compared to low-level restriction cost  *Baseline level = (2.35-0.65) X 23.6% billion AUD/week* | | |
| --- | --- | --- | --- | --- |
|  |  | 80% baseline | Baseline | 120% baseline |
| Daily community transmission during lockdown (*Baseline level =14-day average prior to 11/10/2021*) | 80% baseline | >90%  (8.0) | 90%  (8.5) | 89%  (9.0) |
|  | Baseline | >90%  (9.0) | 90%  (9.6) | 90%  (10.0) |
|  | 120% baseline | >90%  (10.0) | >91%  (10.6) | 90%  (11.1) |

Notes:

1. Outside brackets are the economically justifiable vaccination target for removing the lockdown.
2. Inside brackets is the associated total cost from October 11^th^ 2021, in billion dollars, rounded to the nearest 1-decimal place.

Table G WA: Per capita (AUD and %) cost differences of alternative vaccination rates to the preferred vaccination rate (89%)

|  | Health Care Costs and Welfare Losses | State Border Costs | Total cost |
| --- | --- | --- | --- |
| 70% | +285 (+587.7%) | -84 (-47.5%) | +201 (+89.6%) |
| 80% | +66 (+136.3%) | -40 (-22.5%) | +26 (+11.8%) |
| 90% | -4 (-8.2%) | +4 (+2.5%) | +1 (+0.2%) |

Notes:

1. Numbers are per-capita deviations from the economically justifiable vaccination rate, '+' = higher and '-' =lower
2. Outside brackets are the absolute value of the deviation rounded to the nearest $/person.
3. Inside brackets is the relative deviation in percentage rounded to the nearest 1-decimal place.

Table H WA: Sensitivity of preferred vaccination rate to the effectiveness of public health measures after opening up at reducing community transmission and speed of vaccination

|  | | Daily vaccination rate  (Baseline=11,000/day) | | |
| --- | --- | --- | --- | --- |
|  |  | Baseline | 110% baseline | 120% baseline |
| Effectiveness of reduction in community transmission after opening up (*Baseline level =10%*) | 5% | >90%  (643) | >90%  (639) | >90%  (562) |
|  | 10% | 89%  (597) | 89%  (580) | 89%  (518) |
|  | 15% | 85%  (554) | 85%  (531) | 84%  (478) |

Notes: 1. Outside brackets are the economically justifiable vaccination target for opening up the state border.
2. Inside brackets are the associated total cost from October 11^th^ 2021, rounded to the nearest million dollars.

Table I WA: Sensitivity of preferred vaccination rate to opening up with the probability of seeding and state border closure costs

|  | | Border closure costs  *Baseline =40 million AUD/week* | | |
| --- | --- | --- | --- | --- |
|  |  | 80% baseline | Baseline | 120% baseline |
| Probability of seeding  (*Baseline = 0.5% asymptomatic fully vaccinated passengers after opening border*) | Baseline | 90%  (502) | 89%  (597) | 87%  (688) |
|  | 150% baseline | >90%  (545) | 90%  (644) | 90%  (740) |
|  | 200% baseline | >90%  (576) | >90%  (683) | >90%  (785) |

Notes:

1. Outside brackets are the economically justifiable vaccination target for opening the border.
2. Inside brackets is the associated total cost from October 11^th^ 2021, rounded to the nearest million dollars.
