# Supplementary material for: What vaccination rate(s) minimize total societal costs after ’opening up’ to COVID-19? Age-structured SIRM results for the Delta variant in Australia (New South Wales, Victoria and Western Australia)
Source: PLOS Glob Public Health. 2022 Jun 14;2(6):e0000499. doi: 10.1371/journal.pgph.0000499 (PMC10021844; doi:10.1371/journal.pgph.0000499)
Supplement: Attachment — Submitted filename: 02 Response to editor and reviewers.docx [file pgph.0000499.s006.docx]

**RESPONSES TO EDITOR AND REVIEWERS**

**The Editor**

**Comment:** Please provide separate figure files in .tif or .eps format only, and remove any figures embedded in your manuscript file.  If you are using LaTeX, you do not need to remove embedded figures.

**Response:** We have prepared our figure file in eps format and removed all figures from our manuscript file.

**Comment:** Your manuscript is missing the following sections: Methods and Results Section. Please ensure these are present, and in the correct order, and that any references to subheadings in your main text are correct. An outline of the required sections can be consulted in our submission guidelines here: <https://journals.plos.org/globalpublichealth/s/submission-guidelines>

**Response:** The section has been included in our revised paper.

**Comment:** Please update the completed 'Competing Interests' statement, including any COIs declared by your co-authors. If you have no competing interests to declare, please state "The authors have declared that no competing interests exist". Otherwise please declare all competing interests beginning with the statement "I have read the journal's policy and the authors of this manuscript have the following competing interests:"

**Response:** We have updated the competing-interest statement

**Comment:** If you did not receive any funding for this study, please simply state: “The authors received no specific funding for this work.”

**Response:** We have added an acknowledgement section where we state that we received no specific funding for this work and thanked two reviewers for their insightful comments and suggestions.

**Reviewer #1**

**Comment:** In this study, the authors use an age-structured compartmental model to estimate the health and economic impacts of ending COVID-19 control measures at different levels of vaccine uptake. The study is well presented, with methods and results explained in detail, and I credit the authors for releasing their data and code.

My major critique of the paper is that while the authors use an age-structured model, there is no investigation of the effect of vaccinating different proportions of different age groups and this is not mentioned as a limitation in the discussion. As age is the largest risk factor for severe COVID outcomes, the relative burden of, for example, 10% remaining unvaccinated 65+ is substantially greater than 10% remaining unvaccinated 18-65s. I believe this should be investigated and should be relatively straightforward to implement with your model.

**Response:** Thank you for your supportive comment. We have revised the paper (and the data file) to highlight that elderly people are more vulnerable to COVID, and Australia initially prioritized elderly people in its vaccination program. When approved vaccines (AstraZeneca and Pfizer) became more available, vaccinations were opened to other age groups. On day zero of our model (11/10/2021), all eligible people (12+) can have access to vaccines if they wish.

**Comment:** The discussion section is lacking in explanation of model limitations. No movement back to susceptible from vaccinated or recovered (i.e., no waning) - this appears to be a significant factor in countries which achieved high vaccination rates early e.g., UK, Israel. The authors only consider double vaccinated, which may underestimate vaccine effects from single doses.

**Response:** As you suggested, we have added a paragraph in the discussion section to explain the model limitations. The model does not control for waning vaccine effectiveness (before a booster dose) and which is especially important in countries that achieved high vaccinated rates early, such as the UK and Israel. If supported by data availability, the model can be extended to consider partial vaccination and reinfection. While our model could accommodate a three connected SIRM components: unvaccinated, partially vaccinated, and fully vaccinated; there is no publicly available data on Astra Zeneca and Pfizer rollout for each age group noting that these vaccines have different dose intervals from partially to fully vaccinated (12 weeks and 3 weeks respectively). Our model can also be updated by allowing a fraction of those recovered into susceptible compartments. All of these issues have been made included in the revised manuscript.

Thank you for your helpful comments and suggestions. They are very much appreciated.

**Reviewer #2**

**Comment:** Overall, I found the analysis to be thorough and appropriate. I especially appreciate the range of sensitivity analyses that the authors included. The authors do a good job of situating their findings in the global context, noting that the experience with COVID-19 in Australia is unusual and yet offers valuable insights that may apply more broadly.

I have two main criticisms of the manuscript. First, the treatment of vaccination in the model wasn't fully clear to me. I may have missed it (apologies if so!), but I can't find a definition for the V_i parameter, which is presumably the daily vaccination rate, in Equation (1). I also worry about treating V_i as a constant, since this could lead to non-sensical model solutions (e.g., vaccinating > 100% of the population). Long after the model has been defined, the authors say that they reduce the vaccination rate by 20% once 90% vaccination rates have been achieved; this seems arbitrary and doesn't fix the misspecification issues. I elaborate on these issues further below in the minor comments.

**Response:** Thank you for the helpful comments. Our apologies for not being clear about the assumption behind the vaccination parameter for each age group ($V_{i}$). In the revised manuscript we have included additional explanations and equations to provide greater clarity. As you suggested (in a comment below), we used exponential decay to project the daily vaccination rate to avoid the possibility of vaccinating more than 100% of the population. Some numerical results have changed, but key trends remain unchanged*.*

**Comment:** The second main criticism is that I found the manuscript so long and dense that it took a great deal of effort to pull out the most salient points. The authors have useful things to say here, and I worry that those pearls will get drowned out by the surrounding text. I strongly advocate for the authors to put more of the figures and tables and associated text into a supplement, leaving the main text to describe just the central findings and their importance.

**Response:** Thank you for your comment. As you suggested, we have revised the main text to focus on the most essential information and results. Where appropriate, the main text is linked to Supplementary Sections (labelled the S sections as required by the Journal).

**Comment:** Minor comments: Line 70: The term "zero COVID" felt unnecessary and out of place here. I think of "zero COVID" as a policy aim, rather than an epidemiological phenomenon. I recommend simply cutting this.

**Response:** We have removed the term as you suggested.

**Comment:** Table 1: Could you include the overall totals for each state as well? This would be helpful for interpreting the case counts in your simulation figures.

**Response:** We have added the overall totals for each state. Thank you.

**Comment:** 128-134: V_i should also be defined in this paragraph.

Next: some questions about the treatment of vaccination. As it stands, if V_i is a constant, you'll eventually reach some time where you've vaccinated >100% of the population. How did you account for this? Was there some stopping rule to ensure that you didn't end up with non-sensical simulations? Rather than V_i, you might be able to use a term like - Exp[-V_i t], which with the proper initial conditions will solve out to be a curve that (a) starts at 0 with a slope of V_i and (b) saturates at N as time -> infinity.

208-210: the claim that the "numbers of people being fully vaccinated... are proportional to the number of people waiting for vaccinations" doesn't seem to align with the model equations. For this to be true, I'd expect the vaccination rate to be multiplied by the compartment size.

Also: why is the vaccination term exclusively in the susceptible category? Presumably people who are recovered can also get vaccinated. If you end up with an epidemic that infects >10% of the population before you reach 90% vaccination rates (seems possible), then as the model stands, you'll never reach 90% vaccination rates. This possibility isn't discussed, and I'd like to know how you accounted for it.

Line 263-265: Again, confused by the treatment of vaccination. How was this reduction in vaccination rate implemented in the model?

**Response:** Thank you for your insightful comments. To avoid the possibility of vaccinating more than 100% population, we followed your suggestion of using exponential decay to project the daily vaccination rate AFTER the 90% vaccination target is reached. The decay rate is adapted from the Australian Capital Territory (ACT), a sperate jurisdiction surrounded by New South Wales and which had the most rapid rate of vaccination and has the highest vaccination rate (98.5% of persons aged 12 years and older have received 2 doses) in Australia. The proportion of each age group in the daily vaccination rate is assumed to be proportional to the number of susceptible people in that age group who have not been fully vaccinated.

We revised the paper to acknowledge that the number of cases might grow past 10% of the eligible population during lockdown before the 90% vaccination rate is achieved (and less than 90% of the population were susceptible – assuming no reinfection). For the period for which the model was calibrated, less than 1% of the Australian population had had a positive PCR test. We have followed the ‘full’ (2 doses) vaccination rates and targets announced by Australian federal and state governments by dividing the number of fully vaccinated people by the total eligible population.

**Comment:** Equation 1: a few comments here. First, (here and in all equations), for ordinary differential equations, it's more customary to use a common lower-case 'd' rather than the curly 'd' which usually represents partial derivatives. Also, I'd recommend using a different letter (not 'i', which is used in the numerator) to index the sums in the denominator (also in Equations 2, 5, and 6).

Line 252: Is this time step for the simulation output or for the solving algorithm? If it's for the algorithm, then I worry that the step may be too large. One-day steps could lead to some large numerical errors. How did you check for such errors?

Line 275: How did you calculate confidence intervals from a deterministic ODE model?

**Response:** Thank you, and we have revised the presentation of our equations as you suggested. The differential equations have been converted to the analogous finite-difference equations with a one-day time step to match the frequency of published data on COVID19. This conversion also makes our simulation of stochasticity more transparent and reproducible. In particular, $R_{0}$ used to simulate unmitigated daily transmission is randomised between 4 and 8, and transmission during lockdown (i.e., between day zero and when lockdown measures are removed) is randomised between 0.8 and 1.2 of the 14-day average of the observed transmission before day zero. We simulate 1,460,000 randomised outcomes (1000 rounds times 1460 days) and approximate the confidence intervals from the simulated outcomes.

We have corrected the confusing indices in equations 1, 2, 5, and 6 as you suggested.

**Comment:** line 143: "fixed reduction in community transmission associated with opening up" - this doesn't make sense to me; wouldn't community transmission increase with opening up?

**Response:** We have removed this sentence.

**Comment:** line 251: I think there's a cross-referencing issue here; the equation are 1-8.

**Response:** Our apology. The typo has been fixed in the revised paper.

**Comment:** Fig 2: the caption didn't make sense to me. It seems to me that this is just the number of vaccinated individuals over time.

**Response:** We have removed this redundant figure.

**Comment:** Fig 3 would benefit from a legend describing the colors within the figure itself.

**Response:** We have added color legends for this figure for NSW and other similar figures for VIC and Western Australia case studies, as you suggested.

Thank you very much for your comments and suggestions. They are very much appreciated!
