# Supplementary material for: What vaccination rate(s) minimize total societal costs after ’opening up’ to COVID-19? Age-structured SIRM results for the Delta variant in Australia (New South Wales, Victoria and Western Australia)
Source: PLOS Glob Public Health. 2022 Jun 14;2(6):e0000499. doi: 10.1371/journal.pgph.0000499 (PMC10021844; doi:10.1371/journal.pgph.0000499)
Supplement: Attachment — Submitted filename: 02 Response to reviewers.docx [file pgph.0000499.s007.docx]

Response to editors and reviewers (Second-around revision)

Most comments have been addressed adequately, save for a few. Overall, the authors just need to ensure that they address every element of the reviewers’ comments, and particularly adding the required recommendations to the manuscript text, and not only in their response to the reviewers.

The reviewers’ comments that were not adequately addressed:

**Reviewer #1**

**First-Round Reviewer Comment:** In this study, the authors use an age-structured compartmental model to estimate the health and economic impacts of ending COVID-19 control measures at different levels of vaccine uptake. The study is well presented, with methods and results explained in detail, and I credit the authors for releasing their data and code. My major critique of the paper is that while the authors use an age-structured model, there is no investigation of the effect of vaccinating different proportions of different age groups and this is not mentioned as a limitation in the discussion. As age is the largest risk factor for severe COVID outcomes, the relative burden of, for example, 10% remaining unvaccinated 65+ is substantially greater than 10% remaining unvaccinated 18-65s. I believe this should be investigated and should be relatively straightforward to implement with your model.

**First-Round Author Response:** Thank you for your supportive comment. We have revised the paper (and the data file) to highlight that elderly people are more vulnerable to COVID, and Australia initially prioritized elderly people in its vaccination program. When approved vaccines (AstraZeneca and Pfizer) became more available, vaccinations were opened to other age groups. On day zero of our model (11/10/2021), all eligible people (12+) had access to vaccines if they wished.

**Second-Round Reviewer Response:** Not adequately addressed. Authors have been asked to leverage the age-structured aspect of the model to measure impact of different rates of vaccination by age groups. At least, if they are unable to do this then they must state this **explicitly** in the limitation section in the Discussion and perhaps provide some insights on how this could have impacted on their results.

**Second-Round Author Response:** Thank you for your follow-up comments. We have included two additional paragraphs at the end of the discussion. The paragraphs highlight the limitations of our modelling approach that, in part, arises from inadequate data, and how these limitations could be responded to should the data be available.

In response to the effect of vaccinating different proportions of different age groups, we now explicitly state our assumptions prior to day zero (11 October 2021) of our model. That is, while all 12+people were eligible for vaccination on 11 October 2021, there were substantially different rates of vaccination by age groups. This is because Australian governments priortized older cohorts for vaccination before younger people. If we had realiable daily time-series data on the vaccination rate by each age group from the beginning of vaccination program in February 2021, our model could have been calibrated to simulate epidemiological and economic outcomes under alternative priortization strategies, and to evaluate the impact of age priorization with respect to vaccination.

**First-round Reviewer Comment:** The discussion section is lacking in explanation of model limitations. No movement back to susceptible from vaccinated or recovered (i.e., no waning) - this appears to be a significant factor in countries which achieved high vaccination rates early e.g., UK, Israel. The authors only consider double vaccinated, which may underestimate vaccine effects from single doses.

**Author Response:** As you suggested, we have added a paragraph in the discussion section to explain the model limitations. The model does not control for waning vaccine effectiveness (before a booster dose) and which is especially important in countries that achieved high vaccinated rates early, such as the UK and Israel. If supported by data availability, the model can be extended to consider partial vaccination and reinfection. While our model could accommodate a three connected SIRM components: unvaccinated, partially vaccinated, and fully vaccinated; there is no publicly available data on Astra Zeneca and Pfizer rollout for each age group noting that these vaccines have different dose intervals from partially to fully vaccinated (12 weeks and 3 weeks respectively). Our model can also be updated by allowing a fraction of those recovered into susceptible compartments. All of these issues have been made included in the revised manuscript.

Thank you for your helpful comments and suggestions. They are very much appreciated.

**Second-round Reviewer Comment:** Partially addressed. The reviewer is requesting an explicit paragraph which states their study limitations. Though they address the reviewer directly regarding the issue of waning, they don't add this in their lmitations/discussion as the reviewer requested.

**Second-round Author Response:** In the additional paragraphs at the end of the Discussion, we have explicitly stated that the model does not control for waning vaccine effectiveness.

Thank you again for your follow up comments.

**Reviewer #2**

**First-round Reviewer Comment:** 128-134: V_i should also be defined in this paragraph.

Next: some questions about the treatment of vaccination. As it stands, if V_i is a constant, you'll eventually reach some time where you've vaccinated >100% of the population. How did you account for this? Was there some stopping rule to ensure that you didn't end up with nonsensical simulations? Rather than V_i, you might be able to use a term like - Exp[-V_i t], which with the proper initial conditions will solve out to be a curve that (a) starts at 0 with a slope of V_i and (b) saturates at N as time -> infinity.

208-210: the claim that the "numbers of people being fully vaccinated... are proportional to the number of people waiting for vaccinations" doesn't seem to align with the model equations. For this to be true, I'd expect the vaccination rate to be multiplied by the compartment size.

Also: why is the vaccination term exclusively in the susceptible category? Presumably people who are recovered can also get vaccinated. If you end up with an epidemic that infects >10% of the population before you reach 90% vaccination rates (seems possible), then as the model stands, you'll never reach 90% vaccination rates. This possibility isn't discussed, and I'd like to know how you accounted for it.

Line 263-265: Again, confused by the treatment of vaccination. How was this reduction in vaccination rate implemented in the model?

**First-round Author Response:** Thank you for your insightful comments. To avoid the possibility of vaccinating more than 100% population, we followed your suggestion of using exponential decay to project the daily vaccination rate AFTER the 90% vaccination target is reached. The decay rate is adapted from the Australian Capital Territory (ACT), a sperate jurisdiction surrounded by New South Wales and which had the most rapid rate of vaccination and has the highest vaccination rate (98.5% of persons aged 12 years and older have received 2 doses) in Australia. The proportion of each age group in the daily vaccination rate is assumed to be proportional to the number of susceptible people in that age group who have not been fully vaccinated.

We revised the paper to acknowledge that the number of cases might grow past 10% of the eligible population during lockdown before the 90% vaccination rate is achieved (and less than 90% of the population were susceptible – assuming no reinfection). For the period for which the model was calibrated, less than 1% of the Australian population had had a positive PCR test. We have followed the ‘full’ (2 doses) vaccination rates and targets announced by Australian federal and state governments by dividing the number of fully vaccinated people by the total eligible population.

**Second-round Reviewer Comment:** Partially addressed. They did not address the reviewer’s comment on why the vaccination term is only applied in the susceptible category. If they are unable to do so, they must state this in their limitations and explain how this could possibly affect their results.

**Second-around Reviewer Response:** Thank you for your additional comments. In two additional paragraphs in the Discussion, we have explicitly stated that vaccination is only applied to the susceptible category. If we had data on vaccination rates of people after recovering from COVID-19, and also on the effectiveness of post-recovery vaccination, we could have extended our modelling to include post-recovery vaccination and reinfection by adding a flow from recovery to susceptible compartments. Unfortunately, these data (at least for Australia) are not available so we have noted (as you recommended) the deficiencies of the modelling in these additional paragraphs.

Thank you for your follow up comments.
